# Implications of cerebral/cortical visual impairment on life and learning: insights and strategies from lived experiences

**DOI:** 10.3389/fnhum.2024.1496153

**Published:** 2025-01-03

**Authors:** Rachel G. Bennett, Marguerite E. Tibaudo, Ellen C. Mazel, Nai Y.

**Affiliations:** ^1^Perkins School for the Blind, CVI Center, Watertown, MA, United States; ^2^Perkins School for the Blind, Deafblind Program, Watertown, MA, United States; ^3^Perkins School for the Blind, Educational Programs, Watertown, MA, United States; ^4^Consultant, San Francisco, CA, United States

**Keywords:** cerebral/cortical visual impairment, learning, socialization, navigation, mental health, compensatory skills, CVI lived experiences

## 1 CVI: definition, prevalence, and impact

Cerebral/cortical visual impairment (CVI) is a type of vision problem caused by issues in the brain, not in the eyes themselves. In CVI, the brain has trouble processing visual information due to dysfunction in the visual pathways, which are responsible for interpreting what we see (Kong et al., 2012; Sakki et al., 2018). CVI impacts both higher-order visual processing (e.g., recognition) and lower-order visual processing (e.g., tracking, field deficits, acuity) (Bennett et al., 2020; Fazzi et al., 2007; Pilling et al., 2023). CVI can occur at any stage of life, congenital or due to injury, from many causes, and has a spectrum of manifestations (Bauer et al., 2023; Oakes et al., 2024). It often coexists with ocular conditions, cerebral palsy, developmental delay, epilepsy, neurodevelopmental and genetic conditions (Boonstra et al., 2022; Bosch et al., 2016; Fazzi et al., 2007; Olson et al., 2021; Pilling et al., 2023; Tinelli et al., 2020). CVI impacts visual function and functional vision, and both must be considered in assessment and diagnosis efforts (Pilling et al., 2023).

CVI is the leading cause of pediatric visual impairment that may affect up to 1 in 30 children in mainstream classrooms—yet it remains underdiagnosed (Kong et al., 2012; Teoh et al., 2023; Williams et al., 2021). A recent US prevalence study found at least 180,000 kids with CVI or likely CVI, but less than 20% have a CVI diagnosis (Perkins School for the Blind & McKinsey and Company, 2023). CVI can manifest in ways similar to autism, ADHD, learning disabilities, or other developmental conditions, and therefore often gets missed (Chokron et al., 2021). Children with CVI are at a higher risk of developmental disorders affecting cognitive, learning, motor, and social functioning, and overall reduced health-related quality of life outcomes (Collart et al., 2024; Chokron et al., 2021).

While every lived experience is different, there are common CVI behaviors and traits (Table 1) and emerging study of strategies that may support some with CVI (reduced clutter, use eyes or ears, or wait time) (Chokron et al., 2021; Pilling, 2023). Centering the lived experiences of people with CVI is paramount to understanding how CVI affects daily life, the diverse ways CVI manifests, and the varied compensatory skills used for access. With impacted visual attention and recognition, individuals with CVI are likely to develop a unique representation of what things are, who people are, and the actions, events, and meanings that bring people and things together (Treisman, 2006). Providers and educators need to develop and implement individualized instruction and accommodations that match how the person with CVI perceives and accesses their world.

**Table 1:** Perkins 16 CVI visual behaviors.

Visual attention
Visual recognition
Impact of clutter and crowding
Sensory integration and impact on vision
Impact of motion
Visual field abilities
Impact of color
Form accessibility
Visual guidance of upper limbs
Visual guidance of lower limbs
Access to people
Impact of light
Response interval
Visual curiosity and distance viewing
Appearance of the eyes
Movement of the eyes

From Mazel et al. (2021). “Understanding the CVI Visual Behaviors.” CVINow.org.

## 2 Lived experiences of individuals with CVI

In the past several years, more people with CVI are sharing their stories, which continues to widen our understanding of what CVI is and how it affects everyday life. The authors, whose combined professional and personal backgrounds include TVI, deafblind specialist, special educator, person with CVI, and parent of a child with CVI, reviewed 29 sources (online articles, interviews, videos, and blogs) where 23 individuals with CVI discussed their lived experiences and the compensatory skills (e.g., tactile, auditory, visual) used to access their world. The guiding questions for the review were: How does CVI affect access to learning, navigation, and social interaction? What are strategies and compensatory skills people with CVI use to access these areas? How does CVI impact the perception of objects, people, landmarks, and events?

Using the framework of the Perkins 16 CVI visual behaviors (Table 1), the authors chose statements that reflected manifestations of these behaviors. From there, the authors grouped statements by primary visual behavior knowing that other visual behaviors play a role. For example: “Can't find my parents in a crowd” is primarily access to people, and also reflects clutter, sensory integration (crowd noise), and difficulty with visual attention and recognition. Lastly, the authors grouped the statements into the themes of CVI's impact on learning, navigation, and socialization. Another theme emerged from these accounts—CVI's toll on mental and physical health.

These accounts are a small sample and by no means fully represent the spectrum of CVI experiences—yet they can provide a window into understanding the diverse ways CVI manifests.

### 2.1 Learning

Many factors interrupt visual access to learning for individuals with CVI (Chokron et al., 2021; Zhang et al., 2022). A commonly reported issue was that vision is inconsistent or unreliable. People with CVI shared how difficult it can be to interpret objects and how sometimes they can't trust their vision and are never fully certain what an object is—for example, mistaking a “rough, cold, stiff blanket” for a beloved cat (Nai, 2021b). One stated that “using vision steals all cognitive resources,” so they can only look without using other senses or use another sense without vision (Bennett, 2022d). A middle schooler with CVI stated that his body needs to be in a supportive position to better access his vision and his biggest challenge is when there is “complex visual information, auditory information, or both” (Seif and Bennett, 2024). An adult with CVI described that when she's fatigued, her visual field reduces, she can't recognize what she was just looking at, and she “goes almost blind” (Andrésdóttir et al., 2021). Others detailed how multicolored items “play tricks” on their eyes, clutter deeply impacts item recognition, or they can “recognize things only if they are highly familiar” (Bennett, 2021, 2022e).

In a recent study using images, CVI participants were less accurate in identifying images, required more processing time, and showed significantly greater visual search areas and number of fixations per image (Manley et al., 2023). Several with CVI shared challenges with another two-dimensional form: print text. For example, “Reading is really tiring,” “I can't focus on the words on the page,” and “When I read [print] it feels like my eyes are being pulled out from their centers” (Bennett, 2022b; Marquardt, 2023a). Another individual could not perceive distinct letters and had to rely on the tactile memory of a word shape, so after 5–15 min of this process, visual fatigue set in (Lane-Karnas, 2023). One adult with CVI noted that she cannot read sheet music because “there is too much visual clutter on the page” (Andrésdóttir et al., 2021).

Individuals with CVI reported a range of compensatory skills and strategies to access their learning, including using real objects and manipulatives to support concept development, color-coding to follow math equations or multiple pieces of visual information at once, Braille and tactile graphics, audiobooks, and text-to-speech, and reducing visual clutter (Bennett, 2022a, 2023b; Marquardt, 2023b; Nai, 2022; Seif and Bennett, 2024). Many with CVI naturally create their own workarounds and compensatory skills. Professionals working with students with CVI should support and teach compensatory skills that are aligned with the student's sensory channel use (i.e., vision, hearing, tactile) revealed through formal and informal assessments.

### 2.2 Navigation

Individuals with CVI reported that navigating environments is difficult, unsafe, and often scary. An unfamiliar place can be imperceivable without the support of compensatory skills.

In cluttered, crowded, and busy environments, some individuals with CVI described they “can't see,” “can't find my way” and reported feelings of being overwhelmed and highly stressed (Nai, 2021c; Rastogi-Wilson and Bennett, 2021; Bennett, 2022a,b).

Individuals with CVI commented on difficulty perceiving motion, and that moving cars, bikes, and people are “blurry” (Brossart, 2024; Hamilton et al., 2019). Several used cars as examples: “I can't see moving cars,” “I'm just seeing blur because they are moving too fast,” “I can't tell the speed of the car,” and “I first see the door of the car, then the handle, the window, and only after that, I see the entire car” (Bennett, 2022b; Hamilton et al., 2019; Royal Dutch Visio, 2018).

Individuals with CVI reported difficulty perceiving depth and surface changes, which impacts their ability to navigate steps, sidewalks, shadows, and patterned surfaces. One described that “patterned carpets are a nightmare,” another revealed that escalators cause “anxiety attacks,” and one noted that shadows looked like “holes” or “drop-offs” (Andrésdóttir et al., 2021; Bennett, 2022b; Nai, 2021c).

Individuals with CVI recounted the disruptive effect of temperature, mainly how heat can cause vision to shut down and affect other sensory and body systems. A hot day can cause “zero vision days,” reduced visual field, blurred vision, vision to be “white and washed out,” feeling “sick, fatigued, and dizzy,” and reduced ability to process auditory stimuli (Bennett and Caruso, 2021; Marquardt, 2024). People with CVI encounter temperature problems most likely in uncontrolled outside environments, which may lead to issues with safety and the ability to orient and navigate.

Individuals with CVI reported using various compensatory strategies to support safe navigation, such as previewing unfamiliar places, relying on landmarks to orient, using Google Maps features to plan routes or zoom in on landmarks at a destination, memory of familiar routes, a white cane for tactile detection of obstacles and surfaces changes, a guide dog, and human guide (Andrésdóttir et al., 2021; Bennett, 2022a; Brossart, 2024).

### 2.3 Socialization

Individuals with CVI have difficulty attending to and recognizing faces and understanding facial expressions (Bauer et al., 2023). A young adult with CVI described they can perceive “everything about the person except their faces,” while another stated that when people greet them, “I can't see them clearly” (ABC News Australia, 2022). One individual expressed that it's not just a passive lack of seeing and recognizing faces; they also experience “faces as twisted and contorted,” and the only way to listen is to “tune out visually” (Nai, 2021d).

Individuals with CVI have varied experiences around visual social aspects that can cause social breakdowns, anxiety, and isolation. The impact of clutter, crowding, and noise only increases the difficulties of finding people and accessing conversation and social cues (Bennett, 2022c). One stated “multiple conversations taking place simultaneously creates extra clutter, overwhelming my brain” (Bennett, 2022a). Another explained that “people disappear right in front of me” (Brossart, 2024). Some reported difficulty perceiving and processing another person's emotions and social cues (McDowell, 2019).

Individuals with CVI use compensatory skills to identify people, such as recognizing people by their hair color or distinct features (e.g., glasses, beard) (Liu et al., 2016). **One** individual said, “My mom could change her hair and I couldn't recognize her at all” (Hamilton et al., 2019). Some may also rely on auditory information, the sound of footsteps or the recognition of voices, rather than the visual information that faces provide. In social settings, some with CVI reported they arrive when it's not busy, find “quiet corners,” and avoid discussions in large groups (McDowell, 2019).

### 2.4 Mental and physical health

A thread that weaves throughout each of the above areas is CVI's impact on mental health, stress, anxiety, emotional wellbeing, and physical health. Visual impairments “carry a heavy psychological burden,” where people with vision loss have an increased risk of anxiety and depression (Klauke et al., 2023, p. 3). Children with CVI are at risk for health-related quality of life impairments that encompass psychosocial health, and physical, emotional, social, and school functioning (Collart et al., 2024).

In a panel discussion with four adults with CVI at the Perkins 2024 CVI Conference, a key theme was how CVI was so much more than a visual impairment. All shared that CVI is a “full body experience” that can include migraines, nausea, fatigue, and chronic stress, which can have a long-term impact on health (Baskin, 2024). One adult advocated, “If you look at the medical information about CVI, it's not there: It's a visual condition. In reality, we are experiencing full-body medical issues related to CVI” (Baskin, 2024). One described CVI's impact on daily energy, where people without CVI wake up with 20 spoons, but with CVI you might only start with 10 spoons (Baskin, 2024). Another shared, “Sometimes, I just have to go lay down and be horizontal so that my brain can work” (Baskin, 2024).

Some with CVI discuss the stress and anxiety they live with. In the statements reviewed, many described CVI's emotional toll in daily moments with words like “frightened,” “flight or fight,” “anxious,” “stressful,” “overwhelming,” and “scary.” One adult explained, “CVI has left a hole in my life and identity, as understanding the visual world, in part, integrates a big part of your identity and how you relate to the world and people within, touching every aspect of your life” (Bennett, 2022a).

## 3 Discussion

First-hand accounts from individuals with CVI illustrate the diverse presentations of CVI and how visual access is inconsistent due to many factors (e.g., environment, noise, clutter, temperature, fatigue, stress). Vision use can be difficult, disrupting access to learning, safe navigation, and social interactions, which may cause increased stress, anxiety, and isolation, and very real effects on health and wellbeing.

People with CVI shared a range of compensatory skills used for access to education and daily activities that included auditory, kinesthetic, tactile, and visual skills. There is no one-size-fits all approach. Professionals working with people with CVI must always use a person-centered, holistic approach, and consistently consider the impact of the environment, task, learning materials, and context. Comprehensive assessments (CVI, functional vision, learning media, assistive technology, orientation and mobility), ongoing observation, and collaboration among team members are foundational for implementing accessible instruction and services that support learning, safe navigation, social inclusion, and general wellbeing.

With CVI emerging as a common condition in children, we must listen to people with CVI to inform future areas of research and interventions. Some specific avenues to explore are (1) how CVI impacts quality of life outcomes and the interventions that can help improve these outcomes, (2) non-visual medical issues and long-term health effects of living with CVI, (3) strategies and accommodations to reduce fatigue and anxiety, (4) environmental factors that support access to learning (e.g., lighting, reduced clutter, sound levels), (5) the impact of assistive technology and compensatory techniques on daily functioning, (6) the effect of multisensory instructional approaches and dual media literacy on learning outcomes, (7) how to support inclusion in social contexts, or (8) large prevalence studies to capture the heterogeneity in the CVI population, including associated conditions and socioeconomic risk factors.

## 4 Limitations

The sample of first-hand accounts is small and does not fully represent the heterogeneous CVI population or spectrum of manifestations. This group also included individuals with CVI who can share their stories. The authors acknowledge that a large population of those with CVI have complex needs that may limit their ability to convey their experiences. This review exemplifies the need to further analyze the lived experiences of individuals with CVI to uncover the themes, strategies, and challenges for these individuals through a qualitative and mixed-method analysis.

The authors focused primarily on the visual perception of three-dimensional objects, people, and events. Emerging research and individuals with CVI discuss the difficulty of perceiving two-dimensional visual targets, which was briefly mentioned in this article. CVI's impact on the perception of two-dimensional visual targets is a broad topic requiring further analysis. CVI also impacts many visual processing areas (visualization, visuospatial), and further study is needed to ensure individuals with CVI receive holistic and high-quality assessment and educational programming.
